# Oral candidiasis among African human immunodeficiency virus-infected individuals: 10 years of systematic review and meta-analysis from sub-Saharan Africa

**DOI:** 10.1080/20002297.2017.1317579

**Published:** 2017-06-21

**Authors:** Martha F. Mushi, Oliver Bader, Liliane Taverne-Ghadwal, Christine Bii, Uwe Groß, Stephen E. Mshana

**Affiliations:** ^a^ Department of Microbiology and Immunology, Weill Bugando School of Medicine, Catholic University of Heath and Allied Sciences, Mwanza, Tanzania; ^b^ Institute of Medical Microbiology, University Medical Center, Göttingen, Germany; ^c^ Kenya Medical Research Institute, Center for Microbiology Research, Nairobi, Kenya

**Keywords:** Oral candidiasis, *Candida* colonization, HIV infection, non-*albicans Candida* species, fluconazole resistance, sub-Saharan Africa

## Abstract

Oral candidiasis (OC) is the most common opportunistic fungal infection among immunocompromised individuals. This systematic review and meta-analysis reports on the contribution of non-*albicans Candida* species in causing OC among human immunodeficiency virus (HIV)-infected individuals in sub-Saharan Africa between 2005 and 2015. Thirteen original research articles on oral *Candida* infection/colonization among HIV-infected African populations were reviewed. The prevalence of OC ranged from 7.6% to 75.3%. Pseudomembranous candidiasis was found to range from 12.1% to 66.7%. The prevalence of non-*albicans Candida* species causing OC was 33.5% [95% confidence interval (CI) 30.9–36.39%]. Of 458 non-*albicans Candida* species detected, *C*. *glabrata* (23.8%; 109/458) was the most common, followed by *C*. *tropicalis* (22%; 101/458) and *C*. *krusei* (10.7%; 49/458). The overall fluconazole resistance was 39.3% (95% CI 34.4–44.1%). *Candida albicans* was significantly more resistant than non-albicans *Candida* species to fluconazole (44.7% vs 21.9%; *p* < 0.001). One-quarter of the cases of OC among HIV-infected individuals in sub-Saharan Africa were due to non-*albicans Candida* species. *Candida albicans* isolates were more resistant than the non-*albicans Candida* species to fluconazole and voriconazole. Strengthening the capacity for fungal diagnosis and antifungal susceptibility testing in sub-Saharan Africa is mandatory in order to track the azole resistance trend.

## Introduction

Oral candidiasis (OC) is one of the most common fungal opportunistic infections in immunocompromised individuals [1]. OC occurs in up to 95% of human immunodeficiency virus (HIV)-infected individuals during the course of their illness [2,3], and is a prognostic indicator for acquired immune deficiency syndrome (AIDS) [4,5]. In sub-Saharan Africa, there is an increased prevalence of severe immunocompromised conditions, which is associated with a higher incidence of opportunistic infections [6]. Worldwide, it is estimated that 70% of the HIV-infected individuals living in sub-Saharan Africa [6] are at risk of infection with OC.

OC is mainly caused by *Candida albicans* [7], which accounts for up to 81% of cases among HIV-infected individuals [8]. It is documented that between 17% and 75% of healthy individuals can be colonized by *Candida* species [9,10]. However, non-*albicans Candida* species have been implicated in colonization of the oral cavity, eventually causing infection in 20–40% of immunocompromised individuals [10–12].

The increased prevalence of OC among African HIV-infected individuals ranges from 18% [13,14] to >60% [15–17], and this has resulted in increased use of antifungal agents for both prophylactic and treatment purposes [18]. Furthermore, there is an increasing number of reports of *Candida* species that are resistant to azole antifungal agents [19,20]. This list of resistant species includes *C. krusei*, *C. inconspicua*, and *C. norvegensis*, which are all intrinsically resistant to fluconazole and have been isolated from patients with systemic candidiasis [20,21]. There have also been increased reports of fluconazole resistance in *C. glabrata* isolates, which manifests following the use of azole antifungal agents [19,21]. However, data on the spectrum of *Candida* species and the respective antifungal susceptibility profiles among HIV-infected individuals from sub-Saharan Africa are still limited. This systematic review and meta-analysis aimed to report the incidence of the non-*albicans* species in OC among the HIV-infected African population of sub-Saharan Africa between 2005 and 2015.

## Material and methods

**Figure 1.**
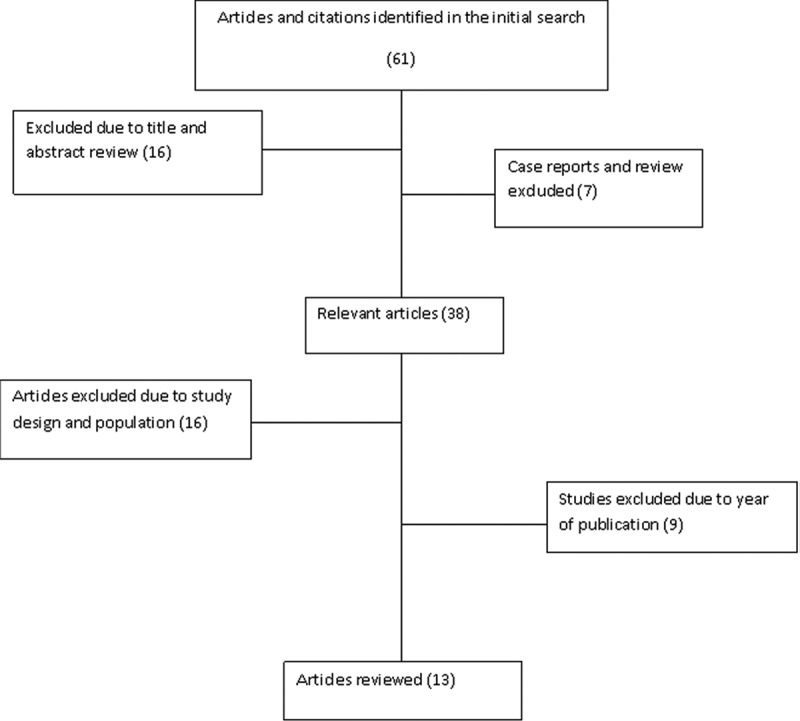
Flowchart showing the literature search and selection criteria.

A literature search of English-language articles undertaking research on oral *Candida* colonization and/or infection was performed using PubMed/MEDLINE, Google Scholar, Web of Knowledge, Google Health, Embase, and POPLINE. The search terms included were ‘oral thrush’, ‘oral candidiasis’, ‘oral *Candida’*, ‘oral *Candida* colonization’, and ‘candidiasis of buccal cavity’, plus African country names in different combinations. New links shown in the abstract were followed to retrieve more abstracts. Thus, a total of 61 abstracts was obtained. All abstracts were carefully reviewed independently by two authors. Sixteen abstracts were excluded since nine were general reports on HIV/AIDS oral manifestations; three were restricted to pediatric populations; and four only described general opportunistic infections, *Candida* infections, or genetic variations of innate immunity and OC. None of the excluded abstracts contained details of oral *Candida* species, pattern of clinical presentation, or antifungal susceptibility. Further analysis excluded one case report and six review articles. The analysis led to 38 articles being obtained on studies on OC that had been conducted in Africa. All 38 articles were carefully reviewed, and a further 25 articles were excluded as they assessed OC among HIV-infected African children or neonates (*n* = 12), were clinical trials (*n* = 2), involved immunocompetent individuals (*n* = 1), comprised a retrospective cohort study (*n* = 1), or had been conducted before 2005 (*n* = 9) (Figure 1). The remaining 13 relevant articles were reviewed independently by two authors. A wide selection of data was extracted from each article and transferred on to a spreadsheet. The data extracted included year of publication, region (country), study population, sampling technique, patient gender, method for *Candida* species identification, use of highly active antiretroviral therapy (HAART), CD4 cell count, prevalence of oral fungal colonization and infection, and the antifungal susceptibility testing scheme.

Data were examined manually and analyzed to obtain the proportion of oral *Candida* colonization and infection. A meta-analysis model was used to calculate the pooled (weighted) proportion of OC, non-*albicans Candida* species, and fluconazole resistance among *C. albicans* and non-*albicans Candida* species. A proportion test was conducted using STATA v.11 to establish the statistical differences between the prevalences of oral *Candida* infection among the HIV-infected African population. A *p* value of <0.05 at a 95% confidence interval (CI) was used to define statistical significance.

### Ethical approval

Ethical clearance for conducting this study was granted by the joint CUHAS/BMC research ethics and review committee, with certificate number CREC/048/2014.

## Results

In total, 13 articles from Nigeria, South Africa, Ethiopia, Uganda, Cameroon, Tanzania, and Ghana were included in this review.

The majority of the articles (*n* = 12; 85.7%) reported on OC, four (28.6%) on both OC and *Candida* colonization, and two (14.3%) only on oral *Candida* species colonization (Table 1).

**Table 1.** Summary of the published articles on oral candidiasis among human immunodeficiency virus (HIV)-infected African populations.

Country	Total	Gender (female)	Method for speciation	Colonization	Infection	95% CI on prevalence	Reference
Ethiopia	215	128	Chromo and tobacco agar, API20C AUX	177 (82.3%)	82 (37.5%)	0.31–0.44	[22]
South Africa	197	197	Chromo agar, germ tube, API20C AUX	117 (59.4%)	18 (9.14%)	0.05–0.13	[23]
Uganda	346	265		Not reported	86 (24.9%)	0.2–0.3	[24]
Tanzania	292	218	Germ tube, AUXACOLOR 2	Not reported	296	–	[25]
Nigeria	300	205	Chromo agar, api20x	75 (0.25%)	Not reported	–	[26]
South Africa	197	197	Chromo agar, api20x	166 (84.3%)	15 (7.6%)	0.89–0.96	[27]
South Africa	212		Chromo agar, germ tube	Not reported	128 (60%)	0.53–0.66	[17]
Cameroon	262		Chromo agar, germ tube	Not reported	126 (40%)	0.34–0.46	[17]
Nigeria	300	158	Chromo agar, api20x	Not reported	120 (60%)	0.54–0.65	[15]
Ghana	267	169	API ID32C	Not reported	201 (75.3%)	0.70–0.80	[16]
Nigeria	213	108	Germ tube, sugar fermentation	Not reported	73 (34.3%)	0.28–0.41	[28]
Uganda	605	469	Chromo agar, API32, PCR	Not reported	316 (52%)	0.48–0.56	[29]
Ethiopia	121	85	Germ tube test and API *Candida*	66 (54.4%)	Not reported	–	[30]

PCR, polymerase chain reaction; CI, confidence interval.

**Figure 2.**
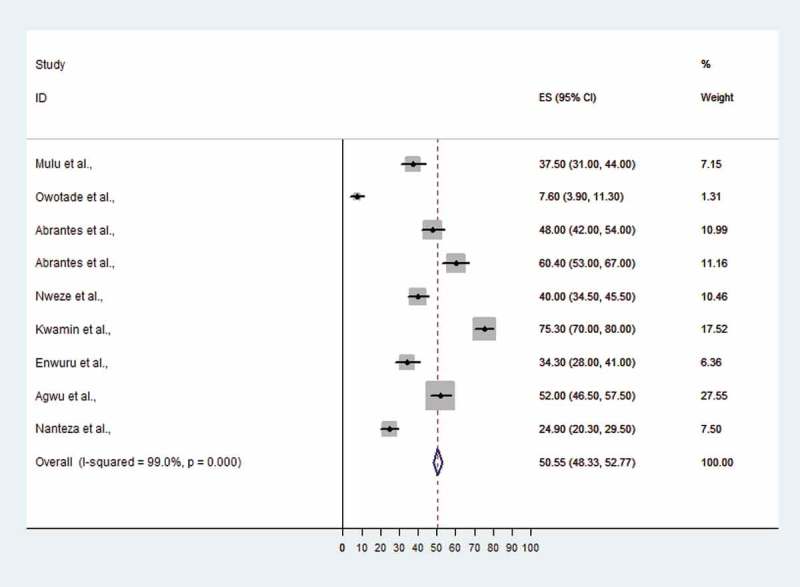
Proportional estimate (ES) with 95% confidence interval (CI) of oral candidiasis (OC) among human immunodeficiency virus (HIV)-infected patients from Africa. The midpoint of each horizontal line segment shows the proportional estimate of OC of each study, while the rhombic mark shows the pooled proportions for all studies.

In six articles that reported on oral *Candida* species colonization among HIV-infected individuals, the prevalence ranged from 0.25% in Nigeria [26] to 82.3% in Ethiopia [22]. With the exception of one article from Tanzania that did not report on OC prevalence [25], the prevalence of oral *Candida* infection was reported to range from 7.6% in Nigeria to 75.3% in Ghana among HIV-infected individuals. The pooled prevalence of OC among HIV-infected Africans was 50.6% (95% CI 48.3–52.8%) (Figure 2). The lowest OC prevalence was detected in South Africa (7.6%, 95% CI 3.9–11.3%) and the highest OC prevalence was observed in Ghana (75.3%, 95% CI 70–80%) (Figure 2).

No clear data were given regarding OC and HIV treatment status. Nine articles involving 2,239 individuals provided data on HIV treatment status. Among the 2,239 individuals, 1,407 (62%) did not receive HAART. Five articles [15,16,26,27,30] had detailed data on OC distribution among individuals receiving and not receiving HAART. Only one article showed that HAART was associated with a significantly lower isolation rate of *Candida* species [26].

**Figure 3.**
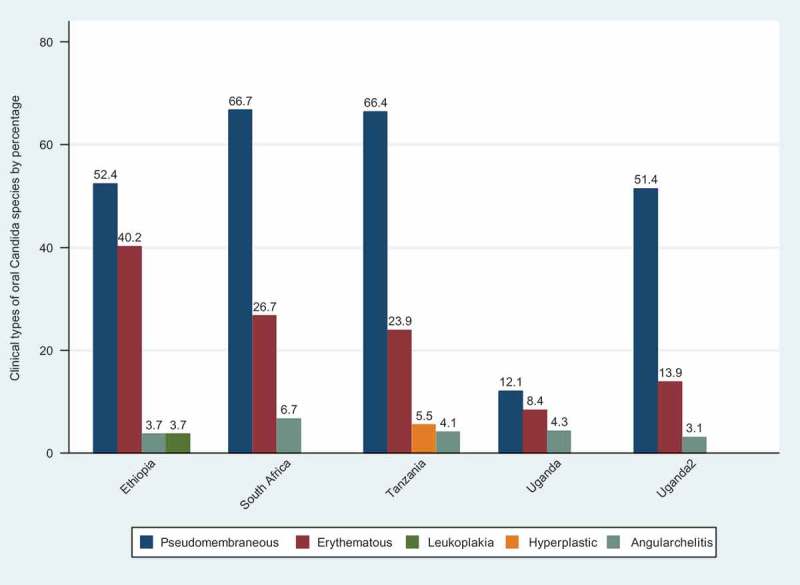
Clinical patterns of oral candidiasis.

Pseudomembranous candidiasis was the most prevalent form of OC reported in these studies. The prevalence of pseudomembranous candidiasis ranged from 12.1% in Uganda to 66.7% in South Africa. The prevalence of erythematous candidiasis (chronic atrophic candidiasis) was highest in Ethiopia, at 40.2%. *Candida* leukoplakia and hyperplastic candidiasis were reported by a single article each, one from Ethiopia and one from Tanzania (Figure 3).

Of 1,795 *Candida* isolates analyzed, *C. albicans* was the most common species (*n* = 1,337; 74.5%, 95% CI 72.2–76.8%), and non-*albicans Candida* species accounted for 458 (25.5%, 95% CI 21.5–29.5%) of isolates. The prevalence of non-*albicans Candida* species colonizing the oral cavity of the immunocompromised African population was found to range from 6.7% to 58.9% in Nigeria.

Of 458 non-*albicans Candida* species detected, *C. glabrata* was the most frequent isolate (23.8%; 109/458), followed by *C. tropicalis* (22%; 101/458) and *C. krusei* (10.7%; 49/458) (Table 2).

**Table 2.** *Candida* species distributions according to different studies.

	Country											
Candida species	Ethiopia	Tanzania	Nigeria	South Africa	Cameroon	South Africa	Nigeria	Ghana	Nigeria	Uganda	Ethiopia	Total
*Candida* detected	223	293	75	116	126	128	120	201	73	316	61	1,795
*C. albicans*	139	250	70	85	92	106	54	139	30	274	53	1,337
	(62.3)	(85.3)	(93.3)	(73.3)	(73)	(82.8)	(45)	(69.2)	(41.1)	(86.7)	(86.9)	
*C. glabrata*	40	20	–	2	24	12	–	2	4	5	–	109
	(17.9)	(6.8)		(1.7)	(19)	(9.4)		(1)	(5.5)	(1.6)		
*C. krusei*	10	10	5(6.7)	1	3	–	2	13	5	–	–	49
	(4.5)	(3.4)		(0.9)	(2.4)		(1.7)	(6.5)	(6.9)			
*C. tropicalis*	27	8	–	7	4	–	22	15	13	5	–	101
	(12.1)	(2.7)		(6)	(3.2)		(18.3)	(7.5)	(17.8)	(1.6)		
*C. dubliniensis*	–	1	–	14	1	10	9	3	–	–	–	38
		(0.3)		(12.1)	(0.8)	(7.8)	(7.5)	(1.5)				
*C. parapsilosis*	–	–	–	1	–	–	18	6	3	2	5	35
				(0.9)			(15)	(3)	(4.1)	(0.6)	(8.2)	
*C. guilliermondii*	–	–	–	–	–	–	11	2	1	–	–	14
							(9.2)	(1)	(1.4)			
*C. sake*	–	–	–	–	–	–	–	5	–	1	–	6
								(2.5)		(0.3)		
*C. kefyr*	–	3	–	–	–	–	2	1	2	–	–	8
		(1)					(1.7)	(0.5)	(2.7)			
*C. famata*	–	–	–	6	–	–	–	2	3	–	–	11
				(5.2)				(1)	(4.1)			
*C. lusitaniae*	–	–	–	–	–	–	2	2(1)	–	–	–	4
							(1.7)					
*C. norvegensis*	–	–	–	–	–	–	–	2	–	4	–	6
								(1)		(1.3)		
Others*	–	1	–	–	–	–	–	6	3	–	–	10
		(0.3)						(3)	(4.1)			
Unidentified	7	–	–	–	2	–	–	–	4	24	3	58
	(3.1)				(1.9)				(5.5)	(7.6)	(4.9)	
Reference	[22]	[25]	[26]	[27]	[17]	[17]	[15]	[16]	[28]	[29]	[30]	

Data are shown as *n* (%).

*Includes Candida spp. reported by single study; 1 C. pintolopesii in Tanzania, 3(4%) C. pseudotropicalis in Nigeria and 1(1%) C.globosa, 1(1%) C. dattila, 1(1%) C. inconspicua, 1(1%) C. hellenica, 1(1%) C. holmii, 1(1%) C. pulcherrima and 1(1%) C. valida in Ghana.

**Figure 4.**
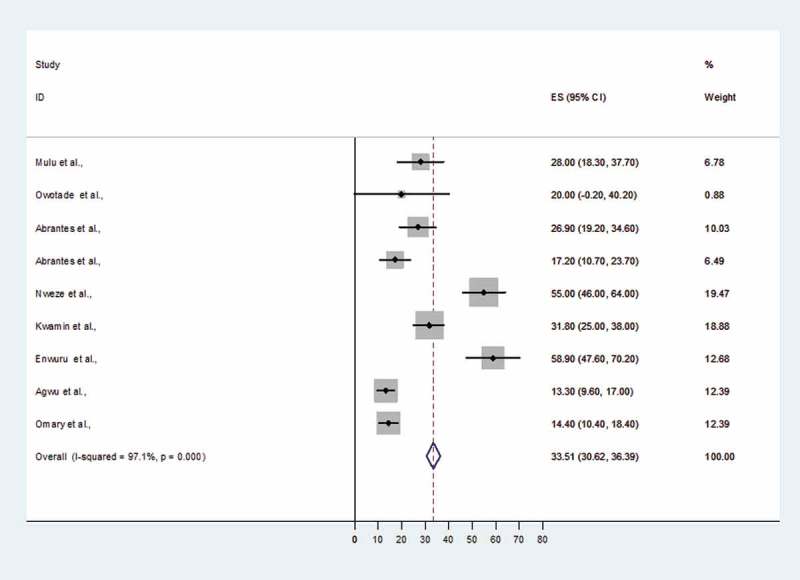
Proportional estimate (ES) of non- *albicans Candida* species causing oral candidiasis (OC) with 95% confidence interval (CI). The midpoint of each horizontal line segment shows the proportional estimate of non-*albicans Candida* species in each study, while the rhombic mark shows the pooled proportions for all studies.

The prevalence of non-*albicans Candida* species causing OC ranged from 13.3% (95% CI 9.6–17%) to 58.9% (95% CI 47.6–70.2%) and both reports involved Nigerian subjects (Figure 4). When the data for non-*albicans Candida* species causing OC among HIV-infected Africans were pooled, the overall prevalence was 33.5% (95% CI 30.9–36.39%) (Figure 4).

Seven articles reported on the occurrence of mixed *Candida* species. Altogether, 1,914 HIV-infected patients were studied, with 236 (12.3%) having mixed *Candida* species. In total, 201 individuals (85.2%) had a mixture of *C. albicans* and a non-*albicans Candida* species.

There were many variations on the breakpoints used in the determination of the antifungal susceptibility. Of the 13 articles analyzed, only five reported on antifungal susceptibility pattern. All five reported the minimum inhibitory concentrations (MICs) by broth microdilution techniques. The Clinical and Laboratory Standards Institute (CLSI) breakpoints were used for interpretation of the drug susceptibility of echinocandins, intraconazole, fluconazole, and amphotericin B (Table 3). One multicenter study undertaken in South Africa and Cameroon [17] used the previously suggested breakpoints for flucytosine [31], voriconazole [32], and posaconale [33] (Table 3). A study conducted in Ethiopia by Mulu et al. [22] used 2 µg/ml as the breakpoint for amphotericin B, as previously reported by Brito et al. [34]. In the study by Mulu et al. [22], the MIC for micafungin was defined as the lowest concentration in which at least 50% of growth of the sample was inhibited.

**Table 3.** Breakpoints for minimum inhibitory concentration determination.

Antifungal agent	Susceptible (µg/mL)	Intermediate (µg/mL)	Resistant (µg/mL)	Source
Fluconazole	≤ 8	16–32	≥ 64	[54]
Itraconazole	≤ 0.12	0.25–0.5	≥ 1	[31,54]
Posaconazole	≤ 0.016	–	≥ 0.016	[33]
Voriconazole	≤ 1	2	≥ 4	[32]
Flucytosine	≤ 4	8–16	≥ 16	[31,54,55]
Amphotericin B	≤ 0.25	–	≥ 1	[31,54,55]
Amphotericin B	≤ 0.25	–	≥ 2	[34]
Caspofungin	≤ 0.25	0.5	≥ 1	[56]
Micafungin	≤ 0.25	0.5	≥ 1	[56]
Anidulafungin	≤ 0.25	0.5	≥ 1	[54,56]

The incidence of fluconazole resistance among *Candida* species was found to range from 5% in Tanzania to 40% in South Africa. The highest rate (13%) of *Candida* species that were resistant to echinocandins (micafungin) was detected in Cameroon (Table 4).

**Table 4.** Antifungal resistance patterns for *Candida albican*s and non-*albicans Candid*a species from different countries.

Country (reference)	Antifungal	Source of breakpoints used	*Candida albicans*				Non-*albicans Candida* spp.			
			Isolates	S (%)	I (%)	R (%)	Isolates	S (%)	I (%)	R (%)
South Africa [17]	Fluconazole	[54]	106	53 (50)	1 (0.9)	52 (49.1)	22	17 (77.3)	4 (18.2)	1 (4.5)
	Itraconazole	[31]		43 (41)	1 (0.9)	62 (58.1)		0	6 (27.3)	3 (13.6)
	Voriconazole	[32]		49 (46)	0	57 (54)		21 (95.5)	0	1 (4.5)
	Amphotericin B	[54]		97 (91.5)	0	9 (8.5)		16 (72.7)	0	6 (27.3)
	Flucytosine	[31]		101 (95.3)	0	5 (4.7)		21 (95.5)	1 (4.5)	0
	Aniladufungin	[54]		101 (95.3)	3 (2.8)	2 (1.9)	12	11 (91.7)	0	1 (8.3)
	Caspofungin	[54]		98 (92.5)	8 (7.5)	0		9 (75)	3 (25)	0
	Micafungin	[54]		106 (100)	0	0		12 (100)	0	0
Cameroon [17]	Fluconazole	[54]	92	45 (49)	1 (0.1)	46 (50)	33	23 (69.7)	7 (21.2)	3 (9)
	Itraconazole	[31]		44 (48)	1 (0.1)	47 (51.8)		9 (27.3)	20 (60.6)	4 (12)
	Voriconazole	[32]		46 (50)	0	46 (50)		31 (93.9)	0	2 (6)
	Amphotericin B	[54]		88 (95.7)	0	4 (4.3)		27 (81.8)	0	6 (18)
	Flucytosine	[31]		86 (93.5)	0	6 (6.5)		31 (93.9)	1 (3)	0
	Micafungin	[54]		92 (100)	0	0	31	10 (32.2)	5 (16)	16 (51.6)
	Aniladufungin	[54]		92 (100)	0	0		23 (74)	5 (16)	3 (9.7)
	Caspofungin	[54]		92 (100)	0	0		23 (74)	7 (22.6)	1 (3)
Nigeria [15,28]	Fluconazole	[54]	84	71 (84.5)	1 (1.2)	12 (14.3)	95	78 (82)	8 (8.4)	9 (9.4)
	Itraconazole	[54]	54	48 (89)	0	6 (11)	60	56 (93.3)	0	4 (6.7)
	Voriconazole	[54]		53 (98)	0	1 (1.8)		59 (98.3)	0	1 (1.7)
	Amphotericin B	[54]		54 (100)	0	0		60 (100)	0	0
	Flucytosine	[31]		49 (90.7)	0	5 (9.3)		55 (91.7)	0	5 (8.3)
Ethiopia [22]	Fluconazole	[54]	25	20 (80)	1 (4)	4 (16)	65	56 (86)	2 (3)	7 (10.7)
	Itraconazole	[54]		20 (80)	3 (12)	2 (8)		57 (87.7)	6 (9.2)	2 (3)
	Micafungin	[56]		24 (96)	0	1 (4)		65 (100)	0	0
	Amphotericin B	[34]		24 (96)	0	1 (4)		65 (100)	0	0
	Flucytosine	[31]		24 (96)	0	1 (4)		65 (100)	0	0
	Ketoconazole	[54]		25 (100)	0	0		62 (95.3)	0	3 (4.6)
Tanzania [25]	Fluconazole	[54]	250	250 (100)	0	0	43	27 (62.8)	0	16 (37.2)
	Itraconazole	[54]		246 (98)	0	10 (4)		28 (65.1)	0	15 (34.9)
	Amphotericin B	[54]		250 (100)	0	0		43 (100)	0	0
	Miconazole	[54]		250 (100)	0	0		43 (100)	0	0
	Nystatin	[54]		250 (100)	0	0		43 (100)	0	0
	Clotrimazole	[54]		250 (100)	0	0		43 (100)	0	0

S, susceptible; I, intermediate; R, resistant.

Among *C. albicans*, micafungin resistance ranged from 0% to 4%, while for non-*albicans Candida* species it ranged from 0% to 51.6% (Table 4). In total, 252 *C. albicans* samples were tested for susceptibility to voriconazole. The resistance rate was found to range from 1.8% to 54.7%, while for non-*albicans Candida* species it ranged from 1.7% to 6% (Table 4). Overall, *C. albicans* was significantly more resistant than non-*albicans Candida* species to voriconazole (104/252 vs 4/115; *p* < 0.001).

**Figure 5.**
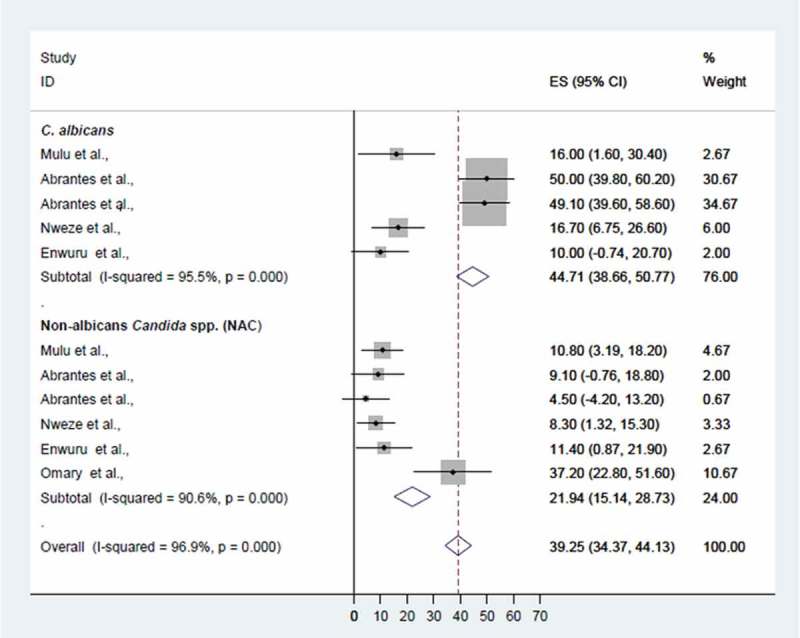
Proportional estimate (ES) of fluconazole resistance by *Candida* species. The midpoint of each horizontal line segment shows the proportional estimate of fluconazole-resistant *Candida* species of each study, while the rhombic mark shows the pooled proportions for all studies by *Candida* species with 95% confidence interval (CI).

When the data for fluconazole resistance were pooled, the overall fluconazole resistance rate was 39.3% (95% CI 34.4–44.1%), while the rate of fluconazole resistance among *C. albicans* was significantly higher than that among non-*albicans Candida* species (44.7%, 95% CI 38.7–50.8% vs 21.9%, 95% CI 15.1–28.7%; *p* < 0.001) (Figure 5).

## Discussion

OC is the leading opportunistic infection among immunocompromised individuals. Sub-Saharan Africa has the world’s highest prevalence of HIV/AIDS patients, with an estimated 24.7 million cases [35]. In the current review, up to 82% of HIV-infected patients were orally colonized by *Candida* species. A similar prevalence has been reported previously in southern India [36,37] and in North America [38]. The overall prevalence in the current review is much higher than that in previous reports from Italy, Brazil, and China [39–41]. The variations in prevalence across the world are considered to be due to differences in diagnostic techniques, geographic and/or ethnic differences, and oral hygiene [38,39].

Oral *Candida* colonization among HIV-infected individuals predicted the subsequent development of OC [7,15,42], mainly owing to the impaired immune system in these patients [43]. In the current review, the highest prevalence of OC among HIV-infected populations was 75%, in Ghana. The incidence of OC was considered relatively stable as it was comparable to a review undertaken between 1984 and 2000 [6].

OC has different clinical presentations with diverse histopathological features [44]. In the current review, pseudomembranous candidiasis (or thrush) was the most common clinical presentation of OC among HIV-infected populations in sub-Saharan Africa. Pseudomembranous candidiasis has also been noted as the most common clinical manifestation of acute OC among immunocompromised individuals in the UK [1,45].

Chronic erythematous candidiasis, which is commonly detected in patients wearing dentures [1], was also commonly found in AIDS patients in a study conducted in Ethiopia by Mulu et al. [22]. This clinical form of OC is characterized by localized chronic erythematous tissues on the dorsum of the tongue, palate, or buccal mucosa [1,46]. Among HIV-infected individuals, erythematous candidiasis is associated with the chronic use of corticosteroids and topical and systemic antibiotics [47]. Its increased prevalence has also been associated with the shedding of the pseudomembranes in persistent or acute pseudomembranous candidiasis [46].

In general, among African HIV-infected individuals, non-*albicans Candida* species contributed about 33.5% of OC. The prevalence of non-*albicans Candida* species was within the range that was observed in Brazil and New Delhi, India [40,48]. As previously documented in Greece, Spain, and New Zealand [18,49,50], the predominant non-*albicans Candida* species detected were *C. glabrata* (24%), *C. tropicalis* (22%), and *C. krusei* (11%). The high prevalence of *C. glabrata* and *C. krusei* among HIV-infected populations from sub-Saharan Africa is of public health importance because of the fluconazole resistance pattern that is normally associated with these species [4,5,51]. Contrary to previous reports from the USA and Finland, where non-*albicans Candida* species were commonly detected in co-infection with *C. albicans* and associated with treatment failure [52,53], in most of the studies in sub-Saharan Africa non-*albicans Candida* species were sensitive to azole and dual presentation was not reported.

The prevalence of non-*albicans Candida* species associated with OC has been linked to a history of fluconazole use [25,28]. However, in the current review, the majority of non-*albicans Candida* species were significantly more sensitive to fluconazole than they were to *C. albicans*. This could be because the non-*albicans Candida* species that are intrinsically resistant to fluconazole contributed only 35% of non-*albicans Candida* species in this review. Therefore, the use of fluconazole may not be the only reason for non-*albicans Candida* species infection. HIV infection with significant depression of the immune system may contribute to the ability of non-pathogenic non-*albicans Candida* species to cause OC in this population.

In Africa, fluconazole is considered to be the drug of choice in both the treatment and prophylactic prevention of fungal infections in HIV-infected individuals and people with AIDS [25,28,57]. The use of fluconazole has been associated with the development of resistance [22,25]. This could explain the observed high rate of fluconazole resistance among *C. albicans*.

It is documented that the overexpression of drug efflux pumps by *C. albicans* due to inappropriate use of azole antifungals leads to the development of resistance to several azole antifungal agents [58,59]. This could explain the high rate of voriconazole resistance among *C. albicans*. However, this mechanism does spare amphotericin B [58], which is expensive and not available in most centers in developing countries. This was confirmed in this review, where the rate of amphotericin B resistance was found to range from 0% to 8.5% among *C. albicans*. With increased inappropriate use of azole antifungal agents [60], resistant strains of *C. albicans* and non-albicans *Candida* species could be selected, underscoring the importance of monitoring antifungal resistance and limiting over-the-counter availability of antimycotic drugs.

Despite the good-quality data summarized in this review, differences in diagnostic techniques and incomplete data reported by most of the studies may have compromised the findings. Most of the studies did not report the HIV disease stage, the use of antiretrovirals, or trimethoprim/sulphamethoxazole prophylaxis. All these factors are known to have an effect on the manifestation of OC.

In conclusion, about one-quarter of the cases of OC among HIV-infected individuals in sub-Saharan Africa are due to non-*albicans Candida* species. In HIV-infected individuals, *C. albicans* was more resistant than non-*albicans Candida* species to fluconazole and voriconazole. There is a need to strengthen the capacity for fungal diagnosis and antifungal susceptibility testing in sub-Saharan African in order to be able to track the resistance trend of *Candida* species in developing countries. Data from these centers will be used to guide the appropriate use of azoles so that they can be preserved for future generations.
